# Human Cerberus Prevents Nodal-Receptor Binding, Inhibits Nodal Signaling, and Suppresses Nodal-Mediated Phenotypes

**DOI:** 10.1371/journal.pone.0114954

**Published:** 2015-01-20

**Authors:** Senem Aykul, Wendi Ni, Washington Mutatu, Erik Martinez-Hackert

**Affiliations:** Department of Biochemistry and Molecular Biology, Michigan State University, East Lansing, Michigan, United States of America; University of Nebraska Medical Center, UNITED STATES

## Abstract

The Transforming Growth Factor-ß (TGFß) family ligand Nodal is an essential embryonic morphogen that is associated with progression of breast and other cancers. It has therefore been suggested that Nodal inhibitors could be used to treat breast cancers where Nodal plays a defined role. As secreted antagonists, such as Cerberus, tightly regulate Nodal signaling during embryonic development, we undertook to produce human Cerberus, characterize its biochemical activities, and determine its effect on human breast cancer cells. Using quantitative methods, we investigated the mechanism of Nodal signaling, we evaluated binding of human Cerberus to Nodal and other TGFß family ligands, and we characterized the mechanism of Nodal inhibition by Cerberus. Using cancer cell assays, we examined the ability of Cerberus to suppress aggressive breast cancer cell phenotypes. We found that human Cerberus binds Nodal with high affinity and specificity, blocks binding of Nodal to its signaling partners, and inhibits Nodal signaling. Moreover, we showed that Cerberus profoundly suppresses migration, invasion, and colony forming ability of Nodal expressing and Nodal supplemented breast cancer cells. Taken together, our studies provide mechanistic insights into Nodal signaling and Nodal inhibition with Cerberus and highlight the potential value of Cerberus as anti-Nodal therapeutic.

## Introduction

The Transforming Growth Factor-ß (TGFß) family ligand Nodal is an essential regulator of vertebrate embryonic development that plays a critical role in formation of the primary body axes and in germ layer specification [1–3]. Beyond embryogenesis, the biological roles of Nodal appear to be limited and, in mammals, Nodal is thought to be largely absent from adult tissues, with exception of some adult stem cell populations and highly dynamic reproductive tissues [4–7]. However, a number of recent studies have shown that Nodal is re-expressed in various metastatic carcinomas, including melanoma and breast cancers, and that Nodal plays a critical role in promoting cancer progression [8–12]. For example, Nodal has been shown to be expressed by aggressive melanoma cells and contributes to their tumorigenicity and plasticity [8], Nodal levels correlate with invasive phenotypes in several breast cancer cell lines [4, 10, 12], and Nodal is significantly overexpressed in tissue samples from patients diagnosed with advanced stage, invasive breast disease [11].

Nodal knockdown, pharmacologic inhibition of Nodal signaling, and Nodal blockade with polyclonal antibodies or with Embryonic Stem Cell (ESC) conditioned medium have been shown to suppress the invasive and tumorigenic phenotype of Nodal expressing, melanoma and breast cancer cells *in vitro* and *in vivo* [4, 8–10, 12–14]. Thus, Nodal is a potential therapeutic target in treatment of melanoma and breast cancers. However, Nodal inhibition is currently not a feasible clinical option, as existing small molecule inhibitors suffer from poor bioavailability and/or inadequate specificity [15, 16], and function-blocking anti-Nodal monoclonal antibodies have yet to be identified.

During fish, frog, chick and mouse embryonic development, Nodal signaling is regulated by the secreted proteins Lefty and Cerberus [1]. Both Lefty and Cerberus co-Immunoprecipitate (co-IP) with Nodal and antagonize Nodal signaling [17–23]. In addition, Lefty blocks Nodal receptor complex formation [17]. Thus, it has been suggested that these embryonic Nodal-signaling antagonists could serve as Nodal inhibitors and potential anti-Nodal therapeutics [24]. Indeed, Lefty purified from stem cell conditioned medium inhibited the colony forming ability of Nodal-expressing human melanoma cells *in vitro* and decreased tumor cell proliferation and increased tumor cell apoptosis when injected into tumors formed from Nodal-expressing human melanoma cells *in vivo* [4]. In contrast to Lefty, the embryonic Nodal antagonist Cerberus is less well understood and its molecular role during development as well as its potential as Nodal inhibitor in cancers have yet to be explored. We therefore undertook to elucidate, using purified, recombinant human proteins, the mechanism of Nodal signaling and Cerberus inhibition, and to characterize biological activities of human Cerberus in several human breast cancer cell lines.

**Figure 1:**
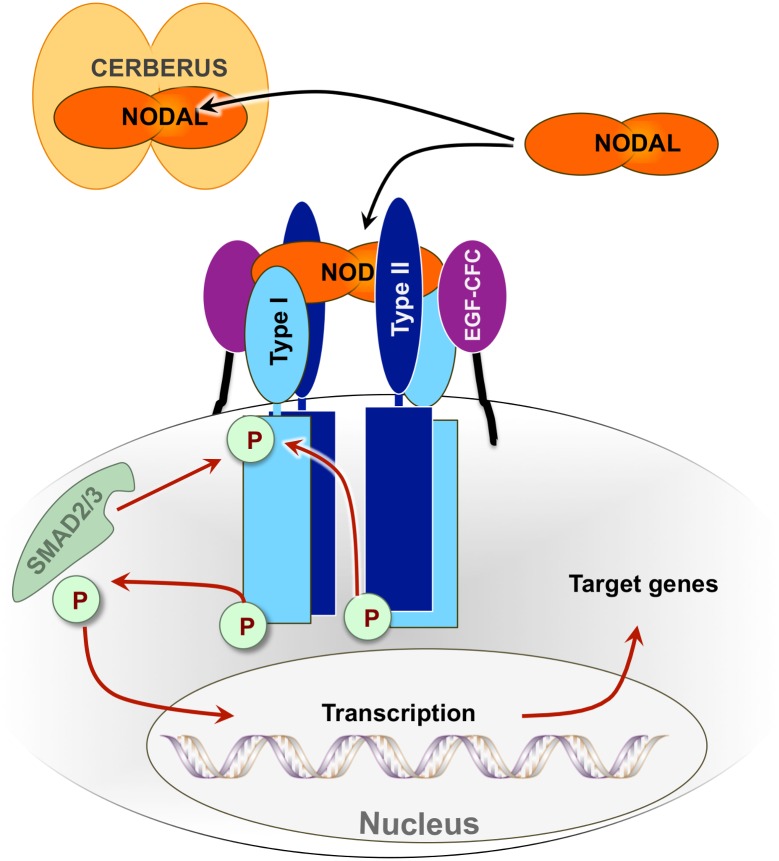
The Nodal signaling pathway. Genetic interaction and co-Immunoprecipitation (co-IP) studies have shown that the TGFß family ligand Nodal (orange) interacts with the extracellular domains of type I Activin receptor-like kinases, including ALK4 and/or ALK7 (light blue), and type II Activin receptor kinases, including ACTRIIA and/or ACTRIIB (dark blue). Simultaneous binding of Nodal to both receptors initiates a phosphorylation cascade that leads to translocation of phosphorylated Smad2 or Smad3 (light green) transcription factors to the nucleus and expression of Nodal target genes. Nodal signaling also requires the EGF-CFC ‘co-receptors’ Cripto-1 and/or Cryptic (purple) during embryonic development. Co-IP studies have shown that EGF-CFC co-receptors interact with Nodal, but how they mediate Nodal signaling is not well understood. The secreted protein Cerberus (light orange) binds Nodal and inhibits Nodal signaling.

Like all members of the TGFß family, Nodal signals by binding the extracellular domains of ‘type I’ and ‘type II’ receptor kinases, thus initiating a phosphorylation cascade that leads to Smad-2/3 mediated expression of Nodal target genes [25–31]. In addition, Nodal signaling during development requires membrane-anchored ‘co-receptors’ [5, 26, 32, 33] (Fig. 1). Here, using human proteins, we identified receptors and co-receptors that associate with Nodal. We showed that Cerberus binds Nodal with high affinity and specificity. We demonstrated that Cerberus blocks binding of Nodal to its receptors and co-receptors, and we showed that Cerberus inhibits Nodal signaling. In addition, we discovered that Cerberus profoundly suppresses aggressive phenotypes in Nodal expressing, human breast cancer cell lines. Taken together, our studies demonstrate that human Cerberus is a specific inhibitor of Nodal and a potential therapeutic for treatment of breast cancers where Nodal plays a role.

## Materials and Methods

### Materials

Recombinant Activin A, GDF-8, GDF-11, GDF1, TGFß-1, BMP-2, BMP-4, BMP-9, AK7-Fc and BMPRII-Fc were obtained from R&D Biosystems or Life Technologies. Activin A was also produced in-house.

### Construction of Expression Plasmids

Synthetic Human Cerberus-hIgg-Fc, human ACTRIIA-hIgg-Fc, human ACTRIIB-hIgg-Fc, human ALK4-hIgg-Fc and human Cryptic-hIgg-Fc genes were obtained from GeneArt. Fusion constructs included full-length Cerberus (1–267), extracellular domains (ECD) of human ActRIIA (1–120), ActRIIB (1–120), ALK4 (1–110), and Cryptic (1–158). Functional domains (Cerberus and ECDs) were linked to human IgG1 Fc via a 22 amino acid long linker containing a TEV cleavage site, a glycine/serine rich region, and a FLAG-tag. Cripto-1 was cloned from cDNA obtained from Thermo Scientific. An amplicon encompassing Cripto1 (1–151) was fused to hIgg1-Fc domain using PCR.

### Purification of Proteins

All proteins were expressed using Chinese Hamster Ovary (CHO) cells. Cerberus-Fc, ACTRIIA-Fc, ACTRIIB-Fc, Cryptic-Fc, and Cripto-1-Fc were purified from condition medium using Protein A capture. Proteins were eluted with 100 mM Glycine pH 3.0 and immediately neutralized with 2 M Tris, pH 9.0. Proteins were futher purified or analyzed by size exclusion chromatography to ascertain monodispersity. Purified proteins were dialyzed into phosphate-buffered saline, pH 7.5 and stored at -20°C. The purity of the proteins was checked with SDS-PAGE under reducing and non-reducing conditions. The Fc portion from recombinant Cerberus-Fc was removed using Tobacco Etch Virus (TEV) protease followed by protein A affinity and size exclusion chromatography.

### Cell Lines

MDA-MB-231 (HTB-26), BT-549 (HTB-122), Hs578t (HTB-125), MCF-7 (HTB-22) and T47D (HTB-133) were were purchased from ATCC by Michigan State University researchers Kathleen Gallo and Chengfeng Yang and made available for these studies [34, 35]. Cell lines were maintained according to ATCC (American Type Culture Collection) culture conditions. Briefly, MDA-MB-231 cells were grown in DMEM/F12 (Thermo Scientific) medium supplemented with 5% fetal bovine serum (FBS) (Corning, 35–010-CV) and 1% penicillin/streptavidin (P/S). MCF-7 and Hs578t cells were maintained in DMEM (Thermo Scientific) medium supplemented with 10% FBS and 1% P/S. BT-549 cells were maintained in RPMI medium (Thermo Scientific) supplemented with 6% FBS and 1% P/S. T47D cells were maintained in RPMI medium supplemented with 10% FBS, 0.2 units/ml bovine insulin (SigmaAldrich, 11070–73–8) and 1% P/S. All cell lines were grown at 37ºC under humidified, 5% CO_2_ atmosphere. Freshly thawed cells were passaged at least three times before performing assays.

### Immunoblotting

Cells were grown to 80% confluency, washed with 1X PBS and grown for an additional 24 h in fresh medium. Protein lysate was prepared by using ice cold RIPA lysis buffer (150mM NaCl, 1% NP40, 0.1% SDS, 50mM Tris pH 8.0, protease inhibitor cocktail (Roche, 11836153001)). Cells were harvested in lysis buffer and stored at -80ºC. Protein concentration of lysates was determined using Bradford. For Western blot, equal amounts of protein were separated on ‘AnykD’ SDS-polyacrylamide gels (Bio-Rad, 456–9035) under reducing conditions and transferred to Hybond-P membranes (GE Healthcare, RPN2020F). Membranes were blocked with 5% dry milk and incubated with primary Nodal antibody (Santa Cruz Biotechnology, sc-28913) at 1:1000 dilution followed by incubation with Horseradish peroxidase conjugated secondary antibody at 1:5000 dilution. For Cerberus immunoblots, purified Cerberus was separated on ‘AnykD’ SDS-polyacrylamide gels and transferred to Hybond-P membranes. Membranes were probed with 1ug/ml primary Cerberus antibody (RnD Systems, AF1075) followed by incubation with Horseradish peroxidase conjugated secondary antibody at 1:1000 dilution. For p-Smad2 immunoblots, ~1.0×10^5^ cells were plated to 24-well plate and grown to 80% confluency in complete media, washed with 1X PBS, starved overnight and grown for an additional 24 h in serum free medium+0.1%BSA with or without 178 nM Cerberus-Fc. For p-Smad2 immunoblots of T47D cells, ~2.0×10^5^ cells were plated to 24-well plate and grown to 80% confluency in complete media, washed with 1X PBS, starved overnight and grown for additional 24 h in serum free medium with or without 39 nM Nodal and/or 17.8 or 178 nM Cerberus. Protein lysate was prepared by using ice cold RIPA lysis buffer (150mM NaCl, 1% NP40, 0.1% SDS, 0.5% sodium deoxycholate, 50mM Tris pH 8.0, 1X ‘Recom ProteaseArrest’ protease inhibitor cocktail (G-Biosciences, 786–436) and 2X ‘PhosphataseArrest’ phosphastase inhibitor cocktail (G-Biosciences, 786–450)). Cells were harvested in lysis buffer and cell lysis supernatant was stored at -80ºC. Protein concentration of lysates was determined using Bradford Assay. Equal amounts of protein were separated on ‘12%’ SDS-polyacrylamide gels (Bio-Rad, 456–1045) and transferred to Hybond-P membranes. Membranes were blocked with 5% BSA and incubated with primary pSmad2 (Cell Signaling, 3108S), Smad2 (Cell Signaling, 5339S) antibodies at 1:1000 dilution and followed by incubation with Horseradish peroxidase conjugated secondary antibody at 1:2000 dilution. WesternBright ECL HRP substrate was used for detection (Advansta, K-12043-D20). Western blots were visualized by exposing the gel to autoradiography film (Denville, E3018) or by using a Bio-Rad ChemiDoc imaging system. Quantification of immunoblots was done by using ImageJ software. Full immunoblots are shown in the supplemental materials section.

### Surface Plasmon Resonance

Receptor-ligand binding affinities were determined by SPR using the Biacore 2000. Anti-human IgG (Fc) antibody was immobilized onto four channels of a CM5 chip using amine coupling chemistry. 200–300 RU of purified Cerberus-Fc, ACTRIIA-Fc, ACTRIIB-Fc, ALK4-Fc or Cripto-1-Fc was captured on the experimental flow channels. A reference channel was monitored to account for nonspecific binding, drift, and bulk shifts. For kinetic analysis, a concentration series of Nodal and other ligands (Activin A, GDF-8, GDF-11, GDF1, TGFß1, BMP-4, BMP-2 and BMP-9) was injected over experimental and control flow channels at 50 μl/min flow rate. Associations were performed for 300 seconds. Dissociation was peformed for 750 and selected concentrations were also performed for 4000 seconds. Only 750 second dissociations are shown in figures. For inhibition analysis 80 nM Nodal was combined with 0 nM, 40 nM or 400 nM Fc-free Cerberus. The pre-assembled Nodal/Cerberus complexes were injected over experimental and control flow channels at 50 μl/min flow rate. After each binding cycle, the antibody surface was regenerated to base line. All experiments were carried out at 25°C. HBS-EPS buffer (0.01 M HEPES, 0.5 M NaCl, 3 mM EDTA, 0.005% (v/v) Tween 20, pH 7.4). Receptors and ligands, except Nodal, were kept in solutions containing 0.5mg/ml BSA (Sigma-Aldrich, A3059). Nodal containing samples were kept without BSA, as the presence of BSA causes rapid inactivation of recombinant human Nodal. Running buffer for all experiments, except those involving Nodal, contained 0.5mg/ml BSA. Sensograms were analyzed by double referencing. To obtain kinetic rate constants, the processed data was fitted to 1:1 Langmuir interaction model with mass transport limitation using Scrubber or BiaEvaluation software. The equilibrium binding constant *K_d_* was determined by calculating the ratio of binding rate constants *k_d_*/*k_a_*. Binding constanst are summarized in Table 1.

**Table 1:** Equilibrium binding and rate constants.

**Ligand**	**Interacting**	***k_a_*(M^-1^s^-1^)**	***k_d_*(s^-1^)**	***K_d_*(nM)**
**Nodal**	ACTRIIA	2.0×10^4^	2.0×10^-3^	100
	ACTRIIB	~4.9×10^4 (est)^	~4.9×10^-4 (est)^	~10 ^(est)^
	BMPRII	3.1×10^5^	4.6×10^-5^	0.149
	ALK4	~4.6×10^4 (est)^	~3.2×10^-4 (est)^	~15 ^(est)^
	ALK7		No Binding	
	Cripto-1	1.0×10^4^	2.6×10^-4^	16
	Cryptic	5.5×10^2^	1.0×10^-3^	2,000^†^
**Activin A**	ACTRIIA	1.1×10^6^	2.5×10^-5^	0.023
	ACTRIIB	1.5×10^6^	2.7×10^-5^	0.018
	ALK4	2.0×10^5^	4.8×10^-4^	2.4
**Nodal**	Cerberus	1.3×10^4^	1.4×10^-5^	1.1
**GDF-11**	Cerberus	1.2×10^3^	0.014	5,800
**BMP-2**	Cerberus	~2.4×10^4^	~0.072	~3,000^†^

(est): Binding rates were calculated by separately fitting association and dissociation rate constants for each concentration and taking the average of the calculated binding rate constants.

†: Binding rates were calculated by fitting each individual concentration and taking the average of the calculated binding rate constants.

### Proliferation

Cells were seeded in complete medium in 96-well plates at a density of ~ 20,000 cells/ml. For Nodal induction, T47D cells were switched after 24 h to serum free medium containing Nodal and/or Cerberus-Fc. For Cerberus inhibition, MCF-7, Hs578t, BT-549, and MDA-MB-231 cells were switched after 24 h to complete medium with 17.8 or 178 nM Cerberus-Fc or no Cerberus control. Viable cell number was determined by measuring cellular ATP using the ViaLight Cell Proliferation and Cytotoxicity Bioassay Kit (Lonza).

### Migration

MCF-7, Hs578t, BT-549, and MDA-MB-231 cells were seeded in an Ibidi insert (Ibidi GmbH, 81176) in complete growth medium. Once cells reached ~ 80% confluence, the insert was removed and medium was replaced with complete medium containing 2.5 µg/ml Mitomycin C and 17.8, or 178 nM Cerberus-Fc or no Cerberus control. Cells were monitored for up to 48 h and images were taken using an inverted microscope with 10X magnification at 0 h and 24 h. Migration was quantified using Vimasis software (Ibidi).

### Invasion

Experiments were performed using a Cultrex 96 Well Basement Membrane Extract (BME) Cell Invasion Assay kit (Trevigen) and medium containing 2.5 µg/ml Mitomycin C. For Nodal induction, ~ 50,000 T47D cells in serum free medium containing 0, or 39 nM (500 ng/ml) Nodal were seeded in the top chamber of the BME coated Boyden chamber plate. Serum free medium containing 0.2 units/ml bovine insulin was added to the bottom chamber. For Cerberus inhibition, about 10,000 MCF-7, Hs578t, BT-549, and MDA-MB-231 cells in serum free medium were seeded in the top chamber of the BME coated Boyden chamber plate. Complete medium was added to the bottom chamber. Top and bottom chamber contained 0 nM, 17.8 nM, or 178 nM Cerberus-Fc and 2.5 µg/ml Mitomycin C. Cell invasion was determined after 24 h by monitoring Calcein-AM fluorescence in the bottom chamber.

### Colony Formation

MCF-7 or MDA-MB-231 cells (~2,500) were resuspended in 0.35% SeaPlaque agarose (Lonza, 50101) in complete medium containing 0 nM, 17.8 nM, or 178 nM Cerberus-Fc and plated on a layer of 0.7% SeaPlaque agarose containing complete medium. Cells were fed twice weekly with complete medium and the corresponding concentrations of Cerberus-Fc. After three weeks, cells were stained with 0.005% crystal violet and images were taken. Cell clusters were quantified using ImageJ software. Colony forming ability was calculated by dividing the number of colonies by the number of initial cells. Percent colony forming ability was determined by dividing the number of colonies formed with Cerberus-Fc relative to no Cerberus-Fc control.

### Reporter Assay

About 50,000 T47D cells in complete medium were seeded in each well of a 96-well plate and grown overnight. The next day, a solution containing 200 ng pSBE4-luc (experimental luciferase reporter plasmid, firefly luciferase), 2 ng pRL-CMV-luc (control luciferase reporter plasmid, renilla luciferase), 24 μl Lipofectamine 2000 (Life Technologies), and 960 μl Opti-Mem (Life Technologies) was prepared and incubated at room temperature for 30 minutes. After incubation, 3840 μl Opti-Mem was added to the transfection solution, cells were washed with 1X PBS, and 50 μl transfection solution was added to each well. Transfection reagent containing medium was removed the following day, cells were washed with 1X PBS, and medium was replaced with serum free RPMI medium containing test proteins. After 16 h incubation at 37°C, luciferase activity was detected with the Dual-Glo Luciferase Assay System (Promega). Relative luciferase units were calculated by dividing firefly luciferase units (FLU) with renilla luciferase units (RLU).

### Statistics

2D cell-based assays with exception of cell migration were performed in quadruplicates and were repeated at least three different times. Colony forming assays were performed in triplicate. Statistical significance was determined using a two-tailed T-test. P values < than 0.05 marked *, P values < than 0.01 were marked **. Cell invasion assays were performed at least three different times.

## Results

### Nodal binds ALK4, BMPRII and Cripto-1

Mouse, frog and fish Nodal have been shown to signal and/or co-IPs with the ‘type I’ Activin receptor-like kinases ALK4 and ALK7, the ‘type II’ receptor kinases ACTRIIA and ACTRIIB, and the co-receptors Cripto-1 and Cryptic [26, 27, 32]. To confirm that the homologous human receptors and co-receptors interact with Nodal, we examined their binding to Nodal using purified human-hIggFc fusion proteins and Surface Plasmon Resonance (SPR). We captured receptor/co-receptor extracellular domain-Fc (ECD-Fc) fusion proteins by immobilizing an anti-hIggFc antibody on a CM5 SPR sensor chip and we injected recombinant Nodal (RnD Systems) over the captured human receptor and co-receptor extracellular domains.

**Figure 2:**
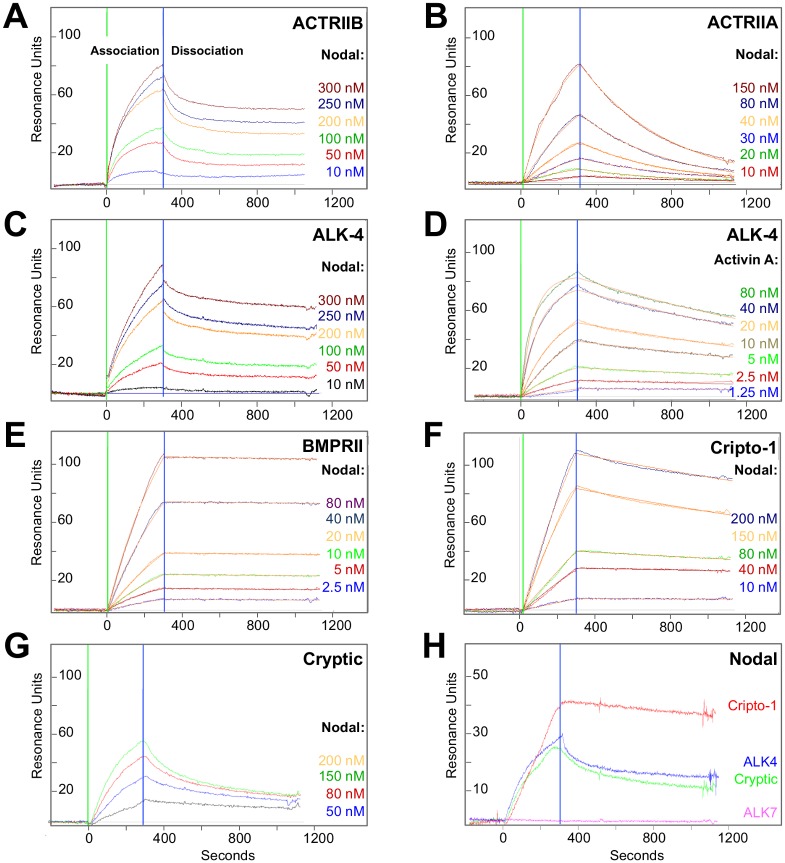
Nodal-receptor interactions. ***(A)*** Nodal binding to ACTRIIB. ACTRIIB-Fc was immobilized on an SPR sensor chip and different concentrations of Nodal were injected as shown. Green vertical bar corresponds to begin of analyte (Nodal) injection (association); the blue vertical bar corresponds to begin of dissociation. The ACTRIIB-Fc-Nodal sensograms could not be fitted to a global kinetic model. Instead, an equilibrium dissociation constant (*K_d_*) of 10 nM was estimated by separately fitting association and dissociation curves. ***(B)*** Nodal binding to ACTRIIA. ACTRIIA-Fc was immobilized on an SPR sensor chip and different concentrations of Nodal were injected as shown. The sensograms fitted a global kinetic model well, giving an association rate constant (*k_a_*) of 2.0×10^4^ M^-1^s^-1^, a dissociation rate constant (*k_d_*) of 2.0×10^-3^ s^-1^, and an equilibrium dissociation constant (*K_d_*) of 100 nM. Fitted curves (orange lines) are superimposed over experimental curves. ***(C)*** Nodal binding to ALK4. ALK4-Fc was immobilized on an SPR sensor chip and different concentrations of Nodal were injected as shown. Like the Nodal-ACTRIIB interaction, Nodal-ALK4 sensograms could not be fitted to a global kinetic model. Instead, an equilibrium dissociation constant (*K_d_*) of 15 nM was estimated by separately fitting association and dissociation curves. ***(D)*** Activin A binding to ALK4. ALK4-Fc was immobilized on an SPR sensor chip and different concentrations of Activin A were injected as shown. The sensograms fitted a global kinetic model very well, giving an association rate constant of 2.0×10^5^ M^-1^s^-1^, a dissociation rate constant of 4.8×10^-4^ s^-1^, and an equilibrium dissociation constant of 2.4 nM. Fitted curves (orange lines) are superimposed over experimental curves. (*E)* Nodal binding to BMPRII-Fc. BMPRII-Fc was immobilized on an SPR sensor chip and different concentrations of Nodal were injected as shown. Like the Activin A-ALK4 sensogram, the Nodal-BMRPII sensograms fitted a global kinetic model very well, giving an association rate constant of 3.1×10^5^ M^-1^s^-1^, a dissociation rate constant of 4.6×10^-5^ s^-1^, and an equilibrium dissociation constant of 0.149 nM. Fitted curves (orange lines) are superimposed over experimental curves. ***(F)*** Nodal binding to Cripto-1-Fc. Cripto-1-Fc was immobilized on an SPR sensor chip and different concentrations of Nodal were injected as shown. The Nodal-Cripto-1 sensograms fitted a global kinetic model, giving an association rate constant of 1.0×10^4^ M^-1^s^-1^, a dissociation rate constant of 2.6×10^-4^ s^-1^, and an equilibrium dissociation constant of 16 nM. Fitted curves (orange lines) are superimposed over experimental curves. ***(G)*** Nodal binding to Cryptic-Fc. Cryptic-Fc was immobilized on an SPR sensor chip and various concentrations of Nodal were injected as shown. The Nodal-Cryptic sensograms were fitted individually and an equilibrium dissociation constant (*K_d_*) of 2,000 nM was calculated by taking the average of association and dissociation rate constants for each binding curve, giving an average association rate constant (*k_a_*) of 5.5×10^2^ M^-1^s^-1^ and an average dissociation rate constant (*k_d_*) of 1.0×10^-3^ s^-1^. Individually fitted curves cannot be displayed. ***(H)*** Comparison of Nodal binding to Cripto-1, Cryptic, ALK4 and ALK7. Equal amounts of Fc fusion proteins as determined by SPR response units were immobilized on the SPR sensor chip and 80 nM Nodal was injected. Cripto-1 (red) shows the best binding, ALK4 and Cryptic (blue and green, respectively) bind Nodal with similar profiles. ALK7 (purple) obtained from RnD Systems and reconstituted as suggested does not bind Nodal.

Our SPR sensograms showed that Nodal binds both ACTRIIB and ACTRIIA (Fig. 2A, B); however, the interaction between Nodal and ACTRIIA is very weak by TGFß-family ligand/receptor standards (*K_d_* ≈ 100 nM). ACTRIIB appears to bind Nodal more stably, as reflected in the slow dissociation of this complex (*k_d_* = 4.9×10^-4^ (s^-1^), Fig. 2A, Table 1), however, we were unable to obtain a satisfactory kinetic model to describe this interaction. Thus, our data indicate that Nodal binds ACTRIIB over ACTRIIA, but neither receptor interacts with Nodal in a manner that is characteristic of cognate TGFß family ligand-type II receptors complexes [36–38]. These unexpected findings led us to ask whether other type II receptors may interact with Nodal in a more characteristic manner. We therefore tested binding of Nodal to the type II TGFß family receptors TGFßRII and BMPRII. We found that Nodal alone does not bind TGFßRII (data not shown). By contrast, we discovered that Nodal binds BMPRII with very high affinity (Fig. 2E, Table 1). This interaction can be described satisfactorily with a standard kinetic model and the resulting binding rate constants are consistent with those determined for other TGFß family ligands and their cognate type II receptors (*k_a_* = 3.1×10^5^ (M^-1^s^-1^), *k_d_* = 4.6×10^-5^ (s^-1^), *K_d_* = 0.15 nM) [36–38].

Our SPR sensograms further revealed that Nodal binds ALK4, but not ALK7 (Fig. 2C, H). The interaction between Nodal and ALK4 (Fig. 2C), however, is not as well defined or as strong as, for example, the interaction between Activin A and ALK4, which fits a standard kinetic model very well (Fig. 2D, Table 1). Concerning co-receptors, Nodal binds strongly the co-receptor Cripto-1, whereas Nodal binding to Cryptic is significantly weaker (Fig. 2F, G, H). Significantly, Nodal binding to Cripto-1 is stable and can be described well with a standard kinetic model (Fig. 2F, Table 1), whereas, the Nodal-Cryptic complex is weak and does not follow well-defined kinetics (Fig. 2G). We calculated binding rates for the Nodal-Cryptic complex by fitting each individual concentration and taking the average of the values. This gave us an estimated *K_d_* of 2,000 nM, approximately two orders of magnitude greater than the analogous Nodal-Cripto-1 equilibrium-binding constant. In summary, we conclude that, in humans, the cognate type II receptor for Nodal is BMPRII and to a lesser degree ACTRIIB (Fig. 2A, E), that the cognate type I receptor for Nodal is ALK4 and not ALK7 (Fig. 2G, H), and that the primary co-receptor is Cripto-1 and not Cryptic (Fig. 2F, G, H). As Cripto-1 has been shown to function and co-IP with ALK4 [5, 26, 39], we also tested binding of Cripto-1 and Nodal-Cripto-1 complexes to ALK4 and ALK7 (S1 Fig.). Intriguingly, using purified human proteins we found that Cripto-1 alone does not bind ALK4 or ALK7, and that Cripto-1 did not enhance or enable the interaction between Nodal and ALK4 (manuscript in preparation).

### Human Cerberus Binds Nodal with High Affinity and Specificity

**Figure 3:**
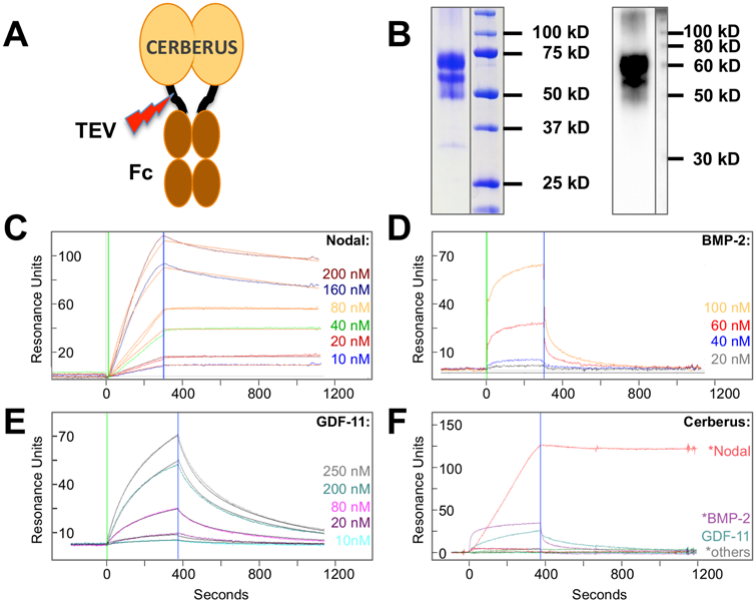
Cerberus ligand binding. ***(A)*** Recombinant Cerberus construct. Full length human Cerberus was fused at the C-terminus to a human Igg1 Fc fragment via a linker containing a TEV cleavage site. ***(B)*** Coomassie Blue stained SDS-PAGE of Cerberus shown on the left side of the panel, Western blot using anti-Cerberus antibody RnD Systems, AF1075 is shown on the right side of the panel. Recombinant Cerberus is purified from Chinese Hamster Ovary cell conditioned medium using protein A capture. Overall, Cerberus-Fc is pure; size heterogeneity may be introduced by variations in glycan structure. The observed smaller Cerberus-Fc fragment could correspond is likely a proteolytic product. We have tested a Cerberus construct that is smaller than the proteolytic product (manuscript in preparation). Nodal binding activity of the shorter Cerberus-Fc is indistinguishable from full-length Cerberus-Fc. ***(C)*** Nodal-Cerberus interaction. Cerberus-Fc was immobilized on an SPR sensor chip and different concentrations of Nodal were injected as shown. The Nodal-Cerberus association constant (*k_a_*) is 1.3×10^4^ M^-1^s^-1^, the dissociation constant (*k_d_*) is 1.4×10^-5^ s^-1^, and the equilibrium dissociation constant (*K_d_*) is 1.0 nM. Fitted curves (orange lines) are superimposed over experimental curves. ***(D)*** BMP-2-Cerberus interaction. Cerberus-Fc was immobilized on an SPR sensor chip and different concentrations of BMP-2 were injected as shown. The sensograms could not be fitted to a global kinetic model due to the extremely fast dissociation rate. Single curve fitting and averaging yielded an estimated BMP-2-Cerberus association rate constant (*k_a_*) of ~2.4×10^4^ M^-1^s^-1^, a dissociation constant (*k_d_*) of ~0.072 s^-1^, and an equilibrium dissociation constant (*K_d_*) of ~3,000 nM. ***(E)*** GDF-11-Cerberus interaction. Cerberus-Fc was immobilized on an SPR sensor chip and different concentrations of GDF-11 were injected as shown. The sensograms were fit to a global kinetic model that yielded an association rate constant of 1.2×10^3^ M^-1^s^-1^, a dissociation rate constant of 0.014 s^-1^, and an equilibrium dissociation constant of 5,800 nM. Fitted curves (grey lines) are superimposed over experimental curves. ***(F)*** Comparison of ligand binding to human Cerberus. Cerberus-Fc was immobilized on an SPR sensor chip and different TGFß family ligands were injected at a concentration of 80 nM as shown. Injections were performed at 40 µl/min. Ligands marked with an asterisk (*), including Nodal, BMP2 and others (Activin A and BMP4), have been shown to interact with Cerberus of different species. Nodal (red) most convincingly binds human Cerberus.

The secreted protein Cerberus is a negative regulator of Nodal signaling during frog and mouse embryonic development and co-IPs with frog Nodal (Xnr1) [18, 22, 23, 40]. To demonstrate that human Cerberus binds human Nodal and inhibits Nodal signaling, we expressed it as fusion protein with human Igg1-Fc (Cerberus-Fc) (Fig. 3A). We purified Cerberus-Fc to homogeneity (Fig. 3B), and examined binding of Nodal to Cerberus by SPR (Fig. 3C). Our sensograms showed that Nodal bound immobilized Cerberus-Fc with low nanomolar affinity (*K_d_* = 1.1 nM, Fig. 3C, Table 1). Its slow dissociation rate (*k_d_* = 1.4×10^-5^ (s^-1^)) indicates that this complex is very stable.

Cerberus from different species has been shown to interact with Nodal and with other TGFß family ligands. For example, functional antagonism, co-IP, or direct binding studies in frog and mouse showed that Cerberus interacts with Bone Morphogenetic Protein (BMP)-2, BMP-4 and/or Activin A [18, 23, 41–43]. We therefore examined by SPR binding of human Cerberus-Fc to these ligands. We also tested binding of TGFß family ligands that interact with ACTRIIB, including GDF-8, GDF-11, and BMP-9. Our sensograms revealed that of all tested TGFß family ligands only BMP-2 and GDF-11 also bound Cerberus-Fc; however, both molecules bound Cerberus-Fc considerably more weakly than Nodal (*K_d_* ≈ 3,000 and 5,800 nM, respectively, Fig. 3D-F). In contrast, human Cerberus-Fc did not bind any other tested TGFß family ligand with consequential affinity, including BMP-4 and Activin A (Fig. 3F). We therefore conclude that human Cerberus binds Nodal with high affinity and specificity.

As others have shown by single SPR injection that BMP-2 stably associates with mouse Cerberus (Cer1) [42], we were surprised to discover that the human Cerberus-BMP-2 complex is not very stable. This can be seen in its fast dissociation rate (*k_d_* = 0.072 (s^-1^), Fig. 3D). To resolve this unexpected observation, we compared the amino acid sequences of Cerberus and several TGFß family ligands and receptors from different species (Table 2). As expected, we found that sequence conservation in TGFß family ligands and receptors is very high throughout a wide range of vertebrate species. For example, mature domains of TGFß family ligands are essentially identical between mice and humans (98–100% sequence identity). By comparison, the primary sequence of Cerberus differs significantly between species and shows only 69% identity between mice and humans. We propose that considerable variations in amino acid sequence could result in unique binding specificities and function of Cerberus in different species.

**Table 2:** Sequence comparison between TGFß family proteins of different species.

	**Mouse**	**Chick**	**Xenopus**	**Zebrafish**
**Cerberus**	69	47 (Caronte)	51	29 (DAND5)
**Nodal**	98	63	60	68 (NR2)
**BMP4**	98	95	96	89
**BMP2**	100	96	96	82
**Activin A**	100	98	87	80
**GDF-11**	100	89 (GDF-8)	99	97
**ACTRIIB**	99	87	77	74
**ALK4**	93	75	65	57
**Cripto-1**	73	49	45	36 (oep)

Mature ligand and receptor/co-receptor ecto domains were compared. Identities were calculated relative to the human proteins over the entire conserved region.

### Human Cerberus Prevents Nodal Interactions and Inhibits Nodal Signaling

**Figure 4:**
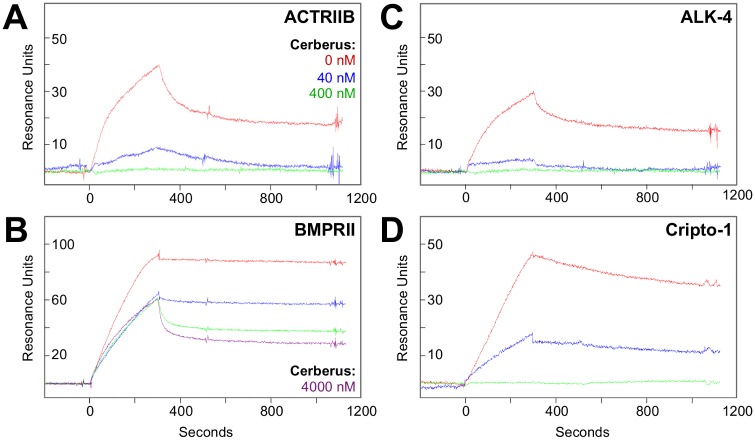
Cerberus inhibition of Nodal interactions. ***(A)*** Cerberus inhibition of Nodal-ACTRIIB binding. ***(B)*** Cerberus inhibition of Nodal-BMPRII binding. ***(C)*** Cerberus inhibition of Nodal-ALK4 binding. ***(D)*** Cerberus inhibition of Nodal-Cripto-1 binding. ACTRIIB-Fc, ALK4-Fc, BMPRII-Fc or Cripto-1-Fc was immobilized on an SPR sensor chip. ***(A-D)*** 80 nM Nodal was preincubated with 0 nM (red), 40 nM (blue), 400 nM (green), or 4000 nM (purple) Cerberus. Preformed Nodal-Cerberus complexes were injected over the sensor chip. ***(A, C, D)*** Cerberus prevents binding of Nodal to ACTRIIB, ALK4, and Cripto-1, as seen in the complete loss of an SPR response at the 400 nM Cerberus concentration (green). Cerberus destabilizes the Nodal-BMPRII interaction, as seen in the altered curve shapes at 4000 nM Cerberus (purple).

Cerberus is an inhibitor of Nodal signaling and Nodal mediated phenotypes during mouse, chick and frog embryonic development [18, 22, 23, 40, 43, 44]. To elucidate the mechanism of Cerberus inhibition, we tested by SPR binding of pre-formed Nodal-Cerberus complexes to Nodal receptors and co-receptors. This format enabled us to identify specific Nodal interactions that Cerberus blocks. We immobilized the Nodal interacting proteins ACTRIIB-Fc, BMPRII-Fc, ALK4-Fc and Cripto-1-Fc on an SPR sensor chip and flowed Nodal or pre-assembled Nodal-Cerberus complexes over the receptor or co-receptor bound sensor chip. In this format, we kept the Nodal concentration constant at 80 nM. Our SPR sensograms revealed that Cerberus effectively blocks binding of Nodal to ACTRIIB, ALK4 and Cripto-1 in a concentration dependent manner (Fig. 4A-D). Cerberus also blocks binding of Nodal to its high affinity receptor BMPRII, however, this inhibition is not as efficient or complete as that of Nodal binding to ACTRIIB (Fig. 4A, B) and appears to be driven primarily by a change in interaction kinetics. To evaluate if Cerberus-Fc is a competitive Nodal inhibitor, we determined the *K_d_* of Nodal binding to ACTRIIB, BMPRII, ALK4 and Cripto-1 in the presence of Cerberus-Fc. We found that for ACTRIIB, ALK4 and Cripto-1, Cerberus-Fc obeys a competitive inhibitor binding-model (S1 Table).

**Figure 5:**
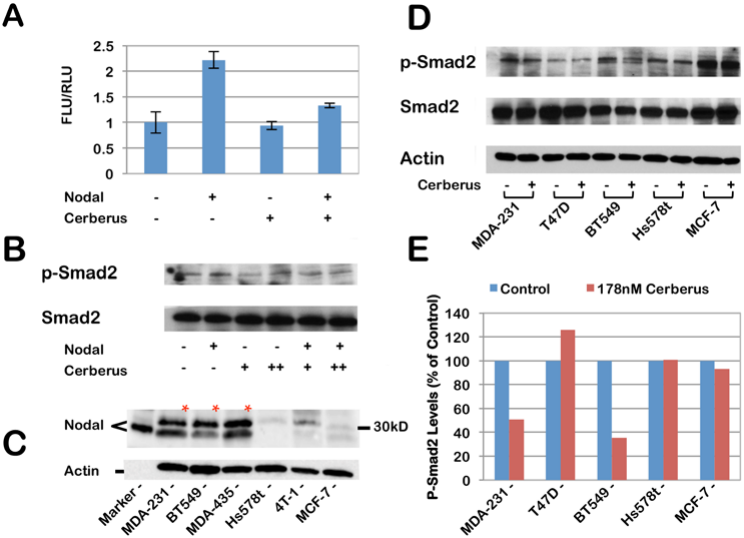
Nodal inhibition with Cerberus in cell lines. ***(A)*** Nodal induced gene expression. T47D cells were transfected with the pSBE4-luc plasmid reporter plasmid and the pRL-CMV-luc plasmid control plasmid. Cells were treated with 39 nM Nodal, and/or 178 nM Cerberus. Nodal signaling was detected by Firefly luciferase activity and normalized against Renilla luciferase activity. ***(B)*** Nodal induced Smad2 phosphorylation. T47D cells were treated with 39 nM Nodal, and/or 17.8 or 178 nM Cerberus. 10 µg of whole cell lysate per lane were loaded on an SDS-PAGE gel and probed with an anti-p-Smad2 antibody. A small increase in Smad2 phosphorylation can be seen when recombinant Nodal is added. Cerberus inhibits Smad2 phosphorylation. (Full blot is shown in S2 Fig.) ***(C)*** Western blot detection of Nodal in five breast cancer cell lines. 10 µg of whole cell lysate per lane were loaded on an SDS-PAGE gel and probed with an anti-Nodal antibody. Hormone receptor positive MCF-7, as well as triple-negative Hs578t, BT-549 and MDA-MB-231 human breast cancer cells were tested. Human MDA-MB-435 melanoma derived cells and mouse 4T-1 breast cancer cells were also probed. (Full blot is shown in S3 Fig.) ***(D)*** Western blot detection of Smad2 phosphorylation in five breast cancer cell lines. Cells were grown in serum free medium with or without 178 nM Cerberus. 5 µg of whole cell lysate per lane were loaded on an SDS-PAGE gel. The gels were probed with an anti-Smad2 antibody. Total Smad2 and Actin were used as controls. (Full blot is shown in S4 Fig.) ***(E)*** Quantitation of p-Smad2 western. The image shown in 5D was scanned and quantitated using ImageJ. Smad-2 phosphorylation is normalized with untreated cells. Both MDA-MB-231 and BT549 cells show a significant decrease in Smad2 phosphorylation when treated with Cerberus. By contrast Hs578t, T47D and MCF-7 Smad2 phosphorylation is not affected by Cerberus.

As binding of Nodal to its cognate receptor kinases has been shown to initiate a phosphorylation cascade that leads to Smad-2/3 mediated gene expression (Fig. 1) [31], we undertook to demonstrate using a Smad-2/3 sensitive reporter gene assay that Cerberus suppresses Nodal mediated gene expression. We transfected T47D human breast cancer cells with pSBE4-luc [45], a Smad-2/3-responsive reporter, and pRL-CMV-luc as control. We treated transfected cells with Nodal and/or Cerberus-Fc to final concentrations of 39 nM and 178 nM, respectively. Our reporter assay showed that Nodal induces Luciferase activity approximately 2.2 fold relative to control and that Cerberus-Fc strongly inhibits the Nodal dependent luciferase signal (Fig. 5A). To demonstrate that this effect is linked with Smad-2/3 phosphorylation, we performed a phospho-Smad2 Western blot (Fig. 5B). We treated T47D cells with 39 nM Nodal and/or 178 nM Cerberus-Fc. Consistent with the reporter assay results, Nodal alone causes a small increase in Smad-2 phosphorylation. Cerberus-Fc alone has no effect on Smad-2 phosphorylation in T47D cells. However, Cerberus-Fc reverses Nodal mediated Smad-2 phosphorylation. Together, our results support the conclusion that Cerberus prevents binding of Nodal to its receptors and co-receptors and thus suppresses Nodal dependent transcriptional programs.

### Nodal Is Expressed in Invasive Breast Cancer Cell Lines and Induces Invasion and Proliferation

Nodal expression has been found to be elevated in invasive and poorly differentiated breast cancer cell lines and Nodal has been shown to promote aggressive breast cancer cell phenotypes [10–13, 46, 47]. To evaluate Cerberus as Nodal inhibitor in human breast cancers, we examined by Western blotting Nodal expression in several human breast cancer cell lines that exhibit different invasive properties *in vitro* and *in vivo*, including MDA-MB-231, BT-549, Hs578t, and MCF-7. In agreement with previous studies, we found that triple negative, highly invasive and metastatic MDA-MB-231 breast cancer cells express high levels of Nodal and that hormone receptor positive, weakly invasive and non-metastatic MCF-7 breast cancer cells express low levels of Nodal (Fig. 5C) [10, 11, 13, 48, 49]. In addition, we found that Nodal is expressed in triple negative, highly invasive and metastatic BT-549 breast cancer cells, in MDA-MB-435 melanoma cells, but not in triple negative, invasive but non-metastatic Hs578t human or in 4T1 mouse breast cancer cells. To test whether Cerberus-Fc can directly impact Nodal mediated Smad-2/3 signaling in Nodal expressing breast cancer cells, we performed a phospho-Smad2 Western blot (Fig. 5D, E). We grew breast cancer cells in serum free medium with or without 178 nM Cerberus-Fc. Consistent with our Nodal Western blot results (Fig. 5C), we found that Cerberus-Fc reduced p-Smad2 levels in Nodal expressing breast cancer cell lines, including MDA-MB-231 and BT549, but not in cell lines where we did not detect a Nodal signal, including T47D, MCF-7 and Hs57t. Quantification of our Western blot results shows that Cerberus-Fc reduces Smad-2 phosphorylation by almost 50% in MDA-MB-231 cells and 75% in BT549 cells.

**Figure 6:**
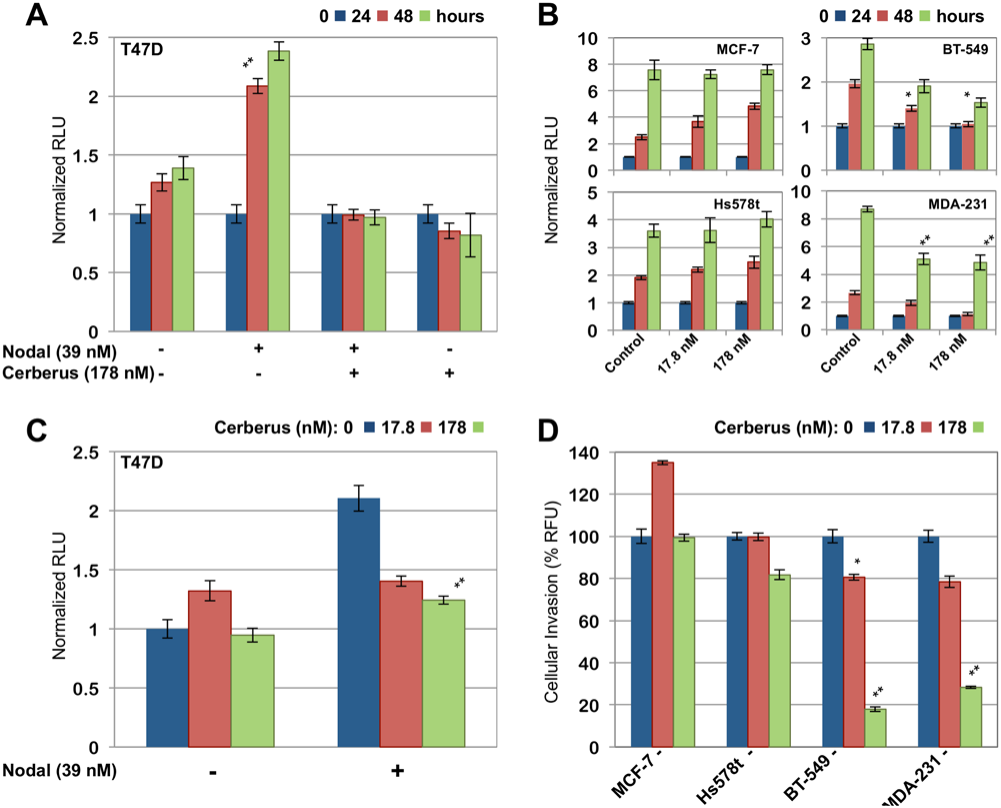
Nodal induces breast cancer cell proliferation and invasion. ***(A)*** Nodal induced cell proliferation. T47D breast cancer cells were grown for 48 h with or without Nodal (39 nM) and/or Cerberus (178 nM). Viable cell number was determined at 0 h (blue), 24 h (red), and 48 h (green) by measuring cellular ATP. ***(B)*** Breast cancer cell proliferation. MCF-7, Hs578t, BT-549, and MDA-MB-231, were grown in the presence of 0 nM (blue), 17.8 nM (red), or 178 nM (green) Cerberus. Viable cell number was determined at 0 h (left), 24 h (middle), and 48 h (right) following addition of Cerberus. ***(C)*** Nodal induced cell invasion. T47D cells were grown for 24 h in a Boyden Chamber. Growth medium contained 2.5 μg/ml Mitomycin C, 0 nM (left) or 39 nM (right) Nodal and 0 nM (blue), 17.8 nM (red), or 178 nM (green) Cerberus. A Cultrex Basement Membrane Extract (BME) coated filter separated the upper and lower chambers. Cell invasion was quantified using Calcein-AM fluorescence. ***(D)*** Cerberus inhibits invasion of Nodal expressing human breast cancer cells. MCF-7, Hs578t, BT-549, and MDA-MB-231, were placed in the top well of Boyden chamber in serum free medium containing 0 nM (blue), 17.8 nM (red) or 178 nM (green) Cerberus and 2.5 μg/ml Mitomycin C. The bottom chamber was filled with matching medium supplemented with serum. The filter separating top and bottom chambers was coated with BME. Cell invasion was quantified using Calcein-AM fluorescence.

To demonstrate that Nodal mediates aggressive phenotypes in breast cancer cell lines, we examined the ability of recombinant Nodal to induce proliferation and invasion in well differentiated, hormone receptor positive T47D human breast cancer cells, which are weakly invasive and express Nodal at low levels [10, 12, 13]. We grew T47D cells in serum free medium lacking Nodal as control or containing recombinant Nodal. We determined the number of viable cells at 0 h, 24 h and 48 h by measuring cellular ATP. We found that recombinant Nodal causes a greater than twofold increase in cell proliferation relative to control (Fig. 6A). In parallel, we performed a transwell invasion assay to characterize Nodal induced cell invasion. We seeded T47D cells supplemented with Mitomycin C in the top chamber of a Boyden chamber plate and we quantified cell invasion through a Basement Membrane Extract (BME) coated filter by measuring Calcein-AM fluorescence in the bottom chamber. We found that 39 nM recombinant Nodal causes an approximately twofold increase in cell invasion relative to control (Fig. 6C). Thus, our results provide further evidence supporting the conclusion that Nodal is expressed in invasive, poorly differentiated human breast cancers, that Nodal can induce breast cancer cell invasion and proliferation, and that Cerberus-Fc inhibits Nodal mediated Smad-2 phosphorylation.

### Cerberus Suppresses Aggressive Phenotypes of Nodal Expressing Breast Cancer Cells

Nodal knockdown and pharmacologic inhibition of Nodal signaling in highly invasive MDA-MB-231 breast cancer cell lines profoundly suppresses invasion and migration [13]. We therefore undertook to evaluate the ability of Cerberus to suppress Nodal mediated cell proliferation, invasion, migration, and colony-forming ability in several human breast cancer cells. For these studies, we selected five human breast cancer cell lines that exhibit an array of characteristics, including invasive and metastatic breast cancer cell lines, which express high levels of Nodal (MDA-MB-231, BT-549), invasive, weakly metastatic cell lines that express low levels of Nodal (Hs578t), as well as non-invasive breast cancer cell lines that express low levels of Nodal (MCF-7, T47D) (Fig. 5C). To demonstrate that Cerberus inhibits proliferation, we measured the number of viable cells at 0 h, 24 h and 48 h following addition of 17.8 nM or 178 nM Cerberus-Fc. Our data showed that Cerberus-Fc profoundly inhibits cell proliferation of Nodal expressing breast cancer cells relative to control samples that were not treated with Cerberus-Fc (Fig. 6B). Indeed, at a Cerberus-Fc concentration of 178 nM we observed greater than 50% inhibition of cell proliferation for MDA-MB-231 and BT-549 relative to control (Fig. 6B). In contrast, Hs578t, MCF-7 and T47D proliferation was not significantly inhibited by Cerberus-Fc (Fig. 6A, B).

To demonstrate that Cerberus suppresses invasion of Nodal expressing breast cancer cells, we characterized cell migration through a transwell filter coated with BME. We plated cells in the top well of a Boyden chamber and incubated cells for 24 h in serum free medium with 2.5 µg/ml Mitomycin C and 0, 17.8, or 178 nM Cerberus-Fc. The bottom chamber contained complete medium as chemoattractant, Mitomycin C and Cerberus-Fc matching the concentration of the top chamber. We assessed invasion by determining the number of cells that invaded the bottom chambers. Our data showed that invasion by the Nodal expressing cell lines MDA-MB-231 and BT-549 is profoundly suppressed by 17.8 nM and 178 nM Cerberus-Fc relative to untreated control samples (Fig. 6D). Indeed, at 178 nM Cerberus-Fc we observed a greater than 70% and 80% reduction in cells that invaded the lower chamber for MDA-MB-231 and BT-549, respectively (Fig. 6D). By comparison, Cerberus-Fc only minimally affected T47D, Hs578t, and MCF-7 cell invasion, resulting in approximately 20% and 0% reductions in invading cells relative to control samples (Fig. 6C, D).

To demonstrate that the observed effects are due to Nodal inhibition by Cerberus, we performed invasion and proliferation assays using the non-invasive T47D breast cancer cell line. We supplemented growth medium with 39 nM recombinant Nodal to induce T47D invasion and proliferation (Fig. 6A, C). In addition, we supplemented growth medium with 17.8 or 178 nM Cerberus-Fc to inhibit Nodal and Nodal mediated phenotypes. Our results showed that Cerberus-Fc suppressed Nodal induced proliferation and invasion in a concentration dependent manner. Indeed, at 178 nM Cerberus-Fc we observed the complete inhibition of Nodal induced proliferation and a substantial reduction in Nodal induced invasion relative to control (Fig. 6A, C).

**Figure 7:**
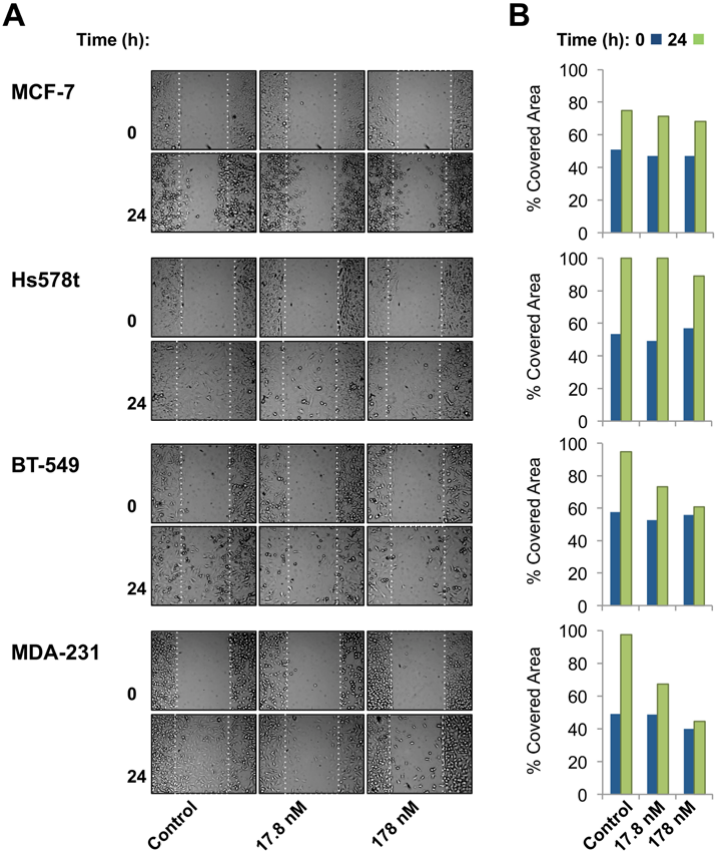
Cerberus inhibits breast cancer cell migration. ***(A)*** Cerberus prevents wound closure of Nodal expressing breast cancer cells. MCF-7, Hs578t, BT-549 and MDA-MB-231 cells were plated in Ibidi culture insert dishes. Cells were grown in complete medium to 80% confluence, inserts were removed to create a gap and medium was replaced with complete medium supplemented with 2.5 μg/ml Mitomycin C and 0 nM (left panel), 17.8 nM (middle panel), or 178 nM (right panel) Cerberus. Images were taken at 0 h and 24 h after removing insert. ***(B)*** Wound closure evaluation. Images taken at 0 h (blue) and 24 h (green) were analyzed using Wimasis software (Ibidi) to quantify cell migration. Graphs within a panel correspond to experiments carried out with 0 nM (left), 17.8 nM (middle), or 178 nM (right) Cerberus.

To show that Cerberus inhibits migration of Nodal-expressing breast cancer cells, we performed a wound-healing assay (Fig. 7, S1 Movie). We plated cells in an Ibidi culture insert. When cells reached 80% confluence, we removed the insert to create a 500 µm gap and replaced medium with medium containing 2.5 µg/ml Mitomycin C and 0, 17.8, or 178 nM of Cerberus-Fc. Our results showed that the invasive cell lines MDA-MB-231, BT-549, and Hs578t completely close the wound within 24 h when grown without Cerberus-Fc (Fig. 7A, B). When grown in medium supplemented with 17.8 nM Cerberus-Fc, the Nodal expressing cell lines MDA-MB-231 and BT-549 did not close the wound. At a Cerberus-Fc concentration of 178 nM, we failed to detect any meaningful wound closure over the course of 48 h (not shown). In contrast, Hs578t and MCF-7 migration was not appreciably affected by Cerberus-Fc and the extent of gap closure did not change relative to untreated control samples.

**Figure 8:**
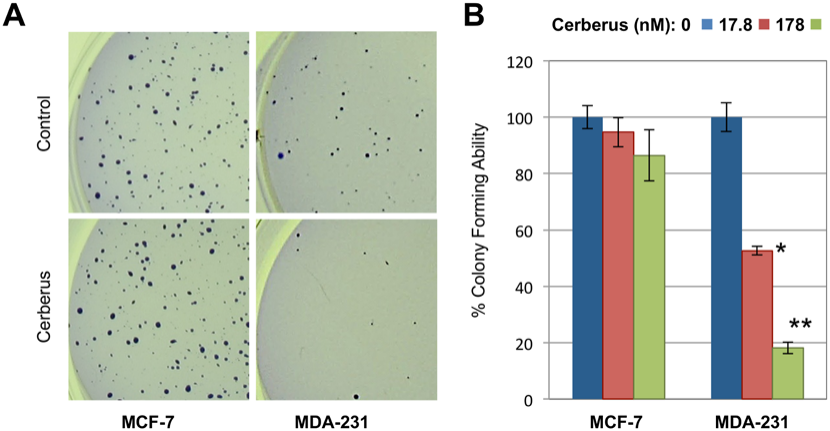
Cerberus suppresses breast cancer cell colony-forming ability. ***(A)*** Representative images of colony formation assay for MCF-7 (left) and MDA-MB-231 cells (right) (10X magnification). Cells were grown in serum containing medium supplemented with 0 nM (top, control), 17.8 nM (not shown) or 178 nM (bottom) Cerberus. ***(B)*** Analysis of colony formation assay for MCF-7 (right) and MDA-MB-231 (left) cells (3A). Images were analyzed using ImageJ to determine number of colonies. Experiments were carried out with 0 nM (blue), 17.8 nM (red), or 178 nM (green) Cerberus. Colony formation assays were performed in triplicates in 6 well plates.

To evaluate the potential *in vivo* response of Nodal expressing breast cancer cells to Cerberus inhibition, we examined the effect of Cerberus-Fc on colony forming ability of two human breast cancer cells, MCF-7 and MDA-MB-231. We grew cells for 3 weeks in agarose containing growth medium lacking or supplemented with 17.8, or 178 nM Cerberus-Fc and determined the number of colonies formed. Our data showed that MDA-MB-231 and MCF-7 cells formed colonies when grown without Cerberus-Fc (Fig. 8A, B); however, the colony forming ability of MDA-MB-231 cells was significantly reduced when cells where grown with Cerberus-Fc. Indeed, less than 20% colonies formed when MDA-MB-231 cells where treated with 178 nM Cerberus-Fc relative to control (Fig. 8A, B). By comparison, the colony forming ability of MCF-7 cells was not changed when cells where grown with 178 nM Cerberus-Fc. Thus, we conclude that Cerberus can suppress proliferation, invasion, migration, and colony-forming ability of Nodal expressing or Nodal supplemented human breast cancer cell lines.

## Discussion

Our goal for this work was to demonstrate that the embryonic Nodal antagonist Cerberus could suppress Nodal-mediated phenotypes. To demonstrate that human Cerberus functionally inhibits Nodal, we first dissected the mechanism of Nodal signaling using quantitative methods and purified human proteins (Figs. 1, 2) [50]. Our studies showed that recombinant Nodal binds the type I Activin receptor-like kinase ALK4, and the co-receptor Cripto-1 as expected (Fig. 2C, F) [50]. The Nodal-ALK4 interaction is weak compared to the analogous Activin A-ALK4 interaction (Fig. 2C, D), but is consistent with the observation that many TGFß family ligands interact with type I receptors with intermediate or low affinities [51]. On the other hand, the putative type I Nodal receptor ALK7, which we obtained commercially, does not bind Nodal as tested and may not directly contribute to Nodal signaling (Fig. 2H). By comparison, Nodal forms a stable complex with the co-receptor Cripto-1 (Fig. 2F). This interaction follows well-defined kinetics, demonstrating that Cripto-1 is a robust Nodal interacting partner. The co-receptor Cryptic, on the other hand, binds Nodal with significantly lower affinity and faster dissociation rates, which would suggests that human Cryptic only plays a minor role in the Nodal signaling pathway in humans. Intriguingly, we did not detect an interaction between the Nodal co-receptor Cripto-1 and ALK4, as has been previously proposed [26, 52–54] (S1 Fig.). It is conceivable that this is because we used human proteins, whereas previous studies used mouse proteins. The two orthologs only share 73% sequence identity and could therefore behave differently (Table 2). However, it is also possible that a bridging interaction between an unidentified TGFß family ligand, Cripto-1 and ALK4 has indirectly led to the previous finding. Indeed, we have conclusive SPR data, which show that a Cripto-1/Nodal complex and a TGFß family ligand/Cryptic complex bind Type I receptors with kinetics that are indistinguishable from those of the free ligand (manuscript in preparation).

Surprisingly, we found that recombinant Nodal does not bind its expected type II receptors ACTRIIA and ACTRIIB with affinities and kinetics that are characteristic of ligand-cognate type II receptor complexes (Fig. 2A, B). Many TGFß family ligands bind their cognate type II receptors with picomolar equilibrium dissociation constants (*K_d_*) and slow dissociation rate constants (*k_d_*) [36–38], whereas Nodal binds ACTRIIA and ACTRIIB with mid nanomolar dissociation constants and fast dissociation rate constants. This suggested to us that ACTRIIA and ACTRIIB are not the dominant, Nodal interacting type II receptors. We therefore tested binding of Nodal to other type II receptors and we discovered that Nodal binds BMPRII with characteristically high affinity and standard kinetics (Fig. 2E). This finding indicates that BMPRII is the dominant Nodal-interacting human type II receptor. Moreover, this finding identifies for the first time a high affinity ligand for BMPRII, which until now has been thought to bind exclusively to BMP ligands with low affinity. While this finding was unexpected, a recent study showed that BMPRII-Fc co-IP’s with Nodal and that BMPRII-Fc inhibits Nodal signaling, while ACTRIIA-Fc and ACTRIIB-Fc do not [55]. That BMPRII has high affinity ligands is of great consequence for the TGFß family and needs to be explored further.

As Nodal has been shown to promote aggressive breast cancer phenotypes and as Nodal knockdown has been shown to reduce tumor incidence and blunt tumor growth in animal models of human breast cancer [8, 10, 11], we undertook to identify and develop a Nodal antagonist that could potentially inhibit Nodal in breast cancers. We hypothesized that the secreted human protein Cerberus could work as Nodal inhibitor, as its frog and mouse homologs co-IP with frog Nodal (Xnr1) and suppress Nodal mediated phenotypes during embryogenesis [18, 22, 23]. To establish the inhibitory activity of human Cerberus, we characterized its interaction with Nodal and its mechanism of inhibition (Figs. 3, 4). We found that human Cerberus forms a stable, high affinity complex with Nodal (Fig. 3C). As frog and mouse Cerberus have been shown to interact with the TGFß family ligands BMP-2, BMP-4, and Activin A [18, 23, 42, 43], we tested binding of human Cerberus to these and several other TGFß family ligands. Significantly, human Cerberus did not appreciably bind any tested TGFß family ligand, indicating that the Nodal-human Cerberus interaction is very specific (Fig. 3F); however, we cannot rule out the possibility that human Cerberus binds other TGFß family ligands that were not tested in this study. Importantly, while our finding that human Cerberus does not bind BMP-2 appears to contradict that obtained for BMP-2 and mouse Cerberus [42], we propose that the difference in binding specificity between Cerberus from these two species is real and can be attributed to the significant variation in amino acid sequence between these two homologs (Table 2). Such sequence dependent functional differences in regulators of embryonic development could underlie one mechanism that led to body plan diversity within mammalian species.

**Figure 9:**
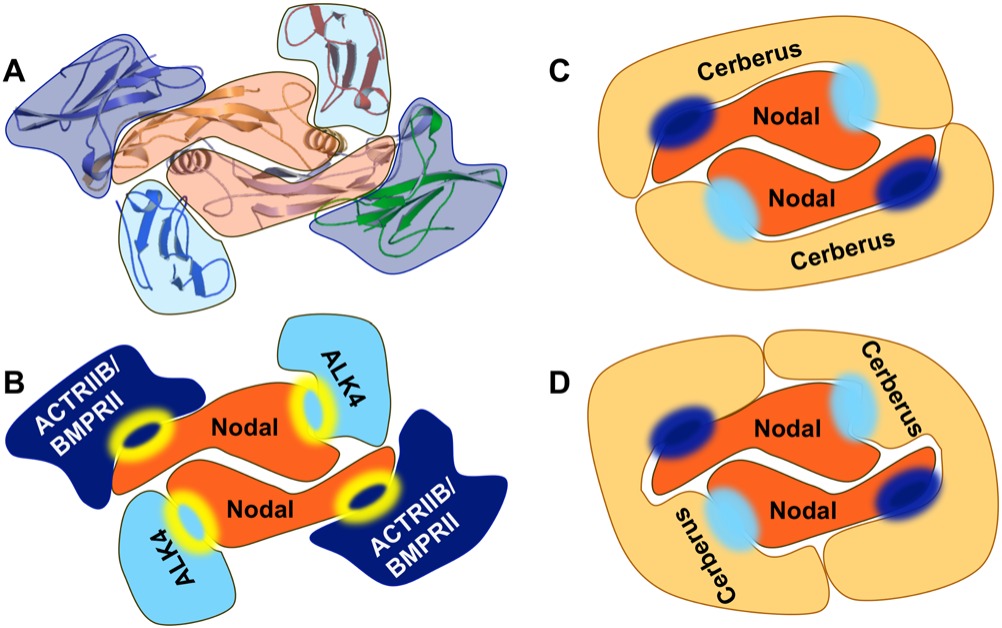
Proposed mechanism of Cerberus inhibition. ***(A)*** Structure of BMP9-ALK1-ACTRIIB complex [37]. The disulfide linked BMP9 homodimer (center, orange) simultaneously binds the extracellular domains of the type I Activin receptor-like kinase ALK1 (light blue) and the type II Activin receptor kinase ACTRIIB (dark blue). ***(B)*** Molecular model of Nodal-receptor interactions based on the BMP9-ALK1-ACTRIIB structure. The disulfide linked Nodal homodimer (center, orange) binds the extracellular domains of the type I Activin receptor-like kinase ALK4 (light blue) and the type II Activin receptor kinase ACTRIIB or BMPRII (dark blue), likely using canonical interaction surfaces (yellow lined circles). ***(C-D)*** Cerberus forms a stable homodimer in solution [58], binds Nodal and prevents binding of Nodal to both receptors. Thus Cerberus blocks simultaneously the ALK4 interaction surface (light blue circle) and the ACTRIIB interaction surface (dark blue circle). We propose that one Cerberus protomer could block interaction surfaces within one Nodal protomer ***(C)*** or two protomers ***(D)***. We expect the overall stoichiometry of this complex to be 2:2.

To elucidate the mechanism of Cerberus antagonism, we examined the ability of Cerberus to inhibit binding of Nodal to its signaling partners. We found that Cerberus blocks binding of Nodal to its receptors ALK4, ACTRIIB and BMPRII, as well as to its co-receptor Cripto-1 (Fig. 4A-D). As structural conservation within the TGFß family indicates that the type I and type II receptor binding sites lie on opposite sides of a Nodal protomer (Fig. 9A, B) [56], our inhibition data suggested that the homodimeric Cerberus could wrap around the Nodal homodimer and cover both receptor interacting surfaces at once (Fig. 9C, D). However, while Cerberus completely blocked Nodal binding to the type II receptor ACTRIIB, Cerberus only appeared to destabilize Nodal binding to the type II receptor BMPRII by lowering the association rate and increasing the dissociation rate (Fig. 4A, B). This suggests that Cerberus is more effective at inhibiting the Nodal-ACTRIIB interaction than the Nodal-BMPRII interaction. While this finding may reflect only the different affinities between Nodal and its type II receptors, it is also possible that BMPRII binds Nodal at a site that is distinct from that of ACTRIIA/ACTRIIB, which would cause BMPRII to behave differently from ACTRIIB [56, 57].

Previous studies showed that Nodal expression is elevated in breast cancer cell lines that are invasive and poorly differentiated, including MDA-MB-231 [10, 11, 13, 48, 49]. Significantly, Nodal knockdown, small molecule Nodal receptor inhibitors and Nodal blockade with an anti-Nodal polyclonal antibody suppresses MDA-MB-231 invasion, proliferation and colony forming ability [10, 11, 13]. As we demonstrated that human Cerberus is a functional Nodal antagonist, we undertook to evaluate its effect on breast cancer cells that express Nodal. We therefore investigated Nodal expression in human breast cancer cell lines that exhibit different characteristics, including triple negative, highly invasive and metastatic MDA-MB-231 and BT-549 cells, triple negative, highly invasive and non-metastatic Hs578t cells, and hormone receptor positive, weakly invasive and non-metastatic MCF-7 cells (Fig. 5C). Based on previous studies [10, 11], we expected to find that Nodal levels are higher in invasive breast cancer cell lines. Indeed, our Western blot data confirmed that Nodal expression is higher in breast cancer cell lines that are invasive and that have the ability to metastasize *in vivo*, whereas Nodal levels are low in weakly or non-invasive and non-metastatic breast cancer cell lines. Within this limited panel of breast cancer cell lines, it appears that Nodal expression is elevated in triple negative breast cancer cells (Fig. 5C) [10].

Next, we examined the effect of Cerberus on Nodal expressing breast cancer cells. We showed that Cerberus-Fc profoundly inhibited proliferation, invasion, and migration of Nodal expressing MDA-MB-231 and BT-549 cells (Figs. 6, 7). In contrast, the effect of Cerberus on breast cancer cells that don’t express Nodal and aren’t induced with recombinant Nodal, including Hs578t, MCF-7 and T47D, is minor (Figs. 6, 7). Similarly, we showed that Cerberus profoundly suppresses the colony forming ability of the Nodal expressing MDA-MB-231 cells, whereas MCF-7 cells are minimally affected (Fig. 8A, B). Significantly, we confirmed that the Cerberus effect is directly related to Nodal inhibition, as Cerberus suppresses invasion and proliferation induced with recombinant Nodal in well-differentiated and weakly invasive T47D cells (Fig. 6A, C). Taken together, our breast cancer cell line studies demonstrated that Cerberus could suppress Nodal-mediated, aggressive breast cancer phenotypes *in vitro*, including in physiologically predictive 3D culture assays, indicating that Cerberus could be an effective inhibitor of Nodal-mediated, aggressive breast cancers *in vivo*.

### Conclusions

We showed that human Cerberus is a functional Nodal inhibitor. Cerberus binds Nodal with high affinity, blocks or reduces binding of Nodal to its interacting partners ALK4, ACTRIIB, BMPRII, and Cripto-1, and thus antagonizes Nodal signaling. Our data further showed that Cerberus could suppress aggressive phenotypes of Nodal expressing breast cancer cell lines. Overall, our studies substantiate Nodal as potential mediator of aggressive breast cancer phenotypes and suggest that Cerberus could work as anti-Nodal therapeutic in breast cancer treatment.

## Supporting Information

S1 Fig Cripto-1 binding to ALK4 and ALK7. (PDF)

S2 Fig Western Blot of Nodal induced Smad-2 phosphorylation in T47D cells. (PDF)

S3 Fig Western Blot of Nodal expression. (PDF)

S4 Fig Western Blot of p-Smad-2 inhibition by Cerberus in breast cancer cells. (PDF)

S1 Movie Supplemental Movie. Migration of MCF-7, Hs578t, BT549 and MDA-MB-231 breast cancer cells in the absence and presence of Cerberus-Fc. Pictures were taken every 5 minutes for approximately 24h using a JuliBr live cell analyzer.
(PPTX)

S1 Table Competitive inhibition analysis. (PDF)
