# Supplementary material for: Human Cerberus Prevents Nodal-Receptor Binding, Inhibits Nodal Signaling, and Suppresses Nodal-Mediated Phenotypes
Source: PLoS One. 2015 Jan 20;10(1):e0114954. doi: 10.1371/journal.pone.0114954 (PMC4300205; doi:10.1371/journal.pone.0114954)
Supplement: S1 Fig — (PDF) [file pone.0114954.s001.pdf]

Figure S1

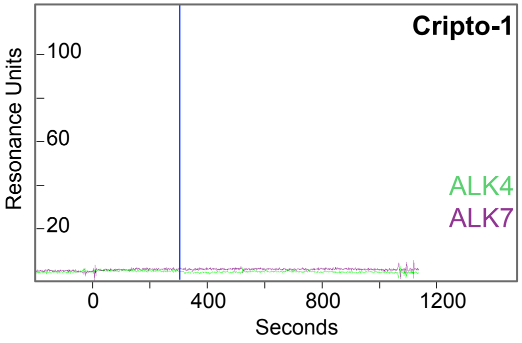

Cripto-1 binding to ALK4 and ALK7. Cripto-1 binding to ALK4 and ALK7. ALK4-Fc and rat ALK7-Fc (RnD systems) were immobilized on an SPR sensor chip and 80 nM Fc free Cripto-1 was injected. Human ALK4 and rat ALK7 do not bind human Cripto-1.
