# Supplementary figures and images for: Human Cerberus Prevents Nodal-Receptor Binding, Inhibits Nodal Signaling, and Suppresses Nodal-Mediated Phenotypes
Source: PLoS One. 2015 Jan 20;10(1):e0114954. doi: 10.1371/journal.pone.0114954 (PMC4300205; doi:10.1371/journal.pone.0114954)

**Figure S2**

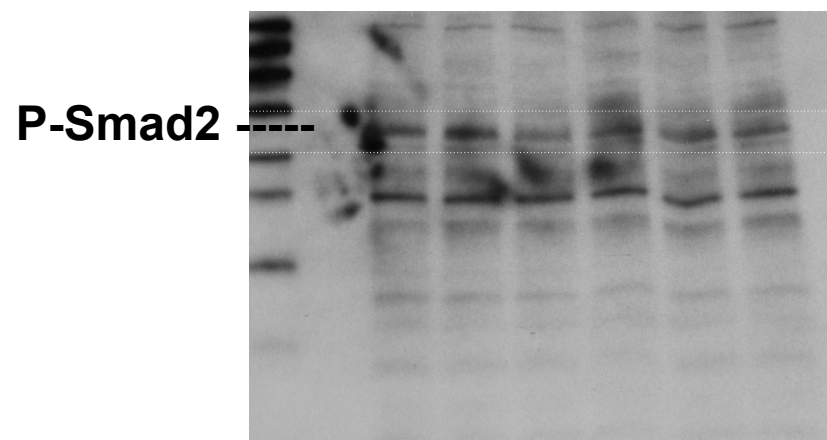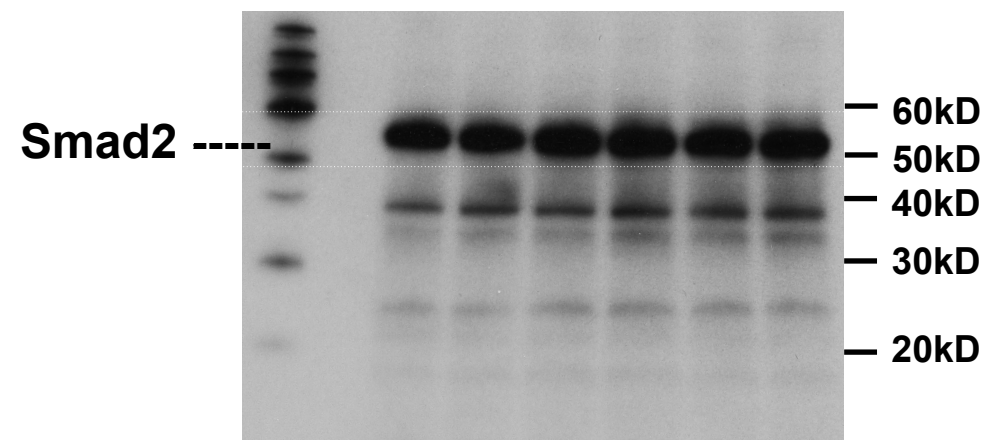

Supplement: S2 Fig — (PDF) [file pone.0114954.s002.pdf]

**Figure S3**

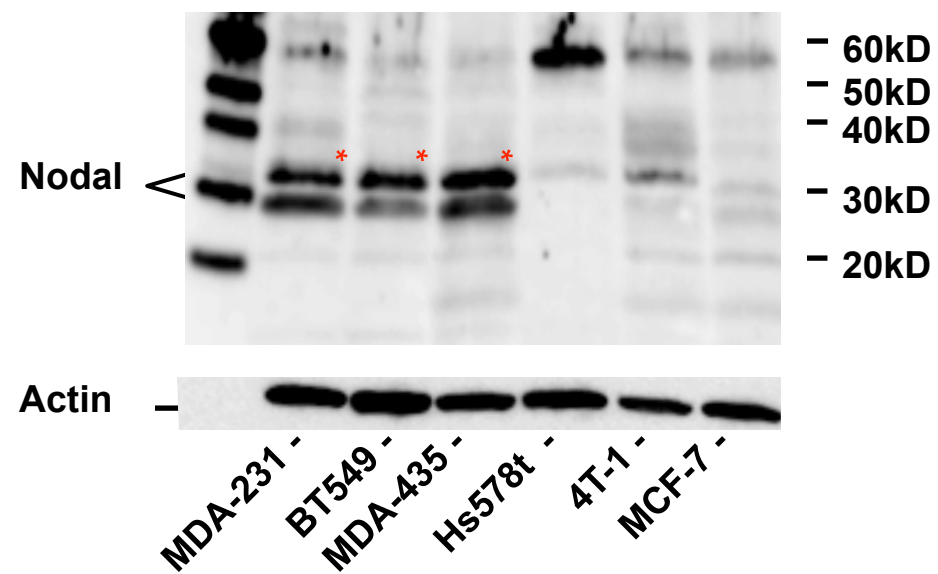

Supplement: S3 Fig — (PDF) [file pone.0114954.s003.pdf]

**Figure S4**

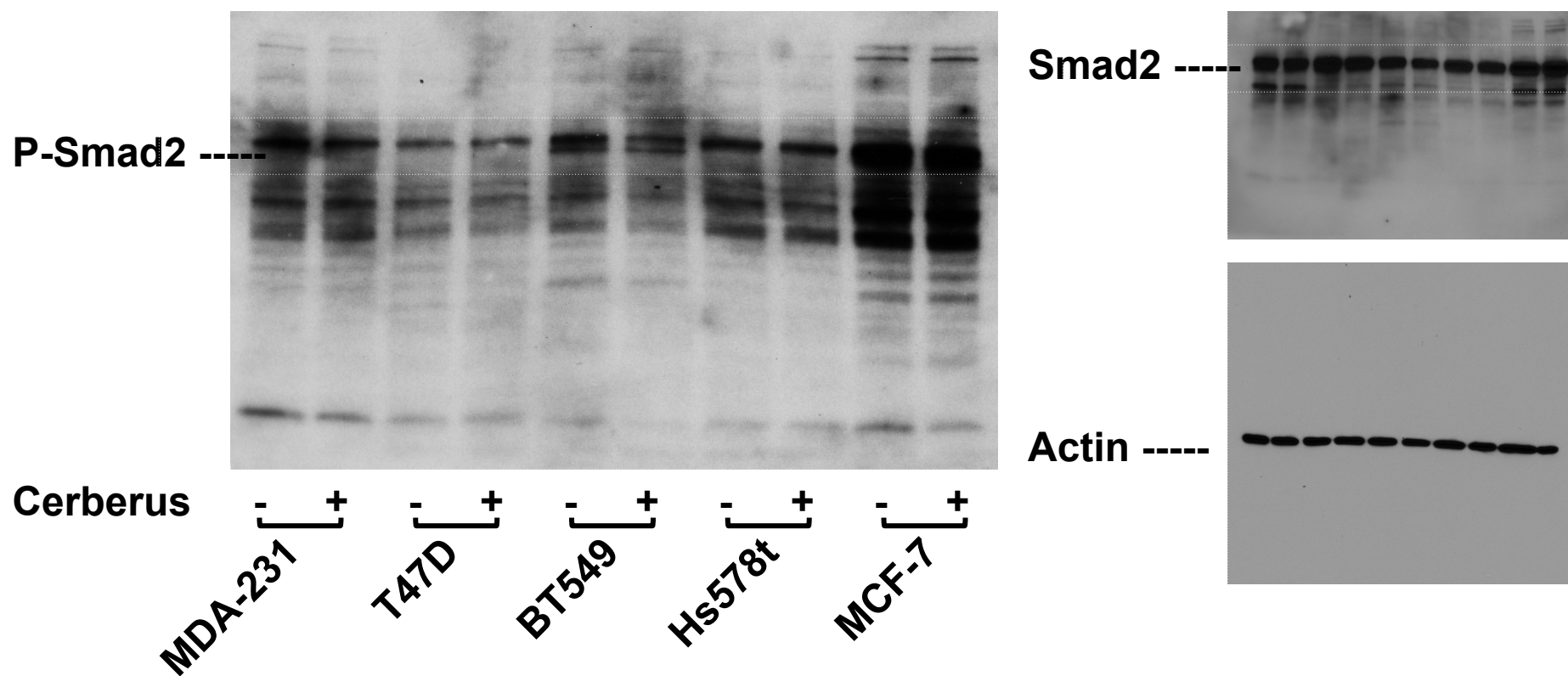

Supplement: S4 Fig — (PDF) [file pone.0114954.s004.pdf]
