# Supplementary material for: Human Cerberus Prevents Nodal-Receptor Binding, Inhibits Nodal Signaling, and Suppresses Nodal-Mediated Phenotypes
Source: PLoS One. 2015 Jan 20;10(1):e0114954. doi: 10.1371/journal.pone.0114954 (PMC4300205; doi:10.1371/journal.pone.0114954)
Supplement: S1 Movie — Migration of MCF-7, Hs578t, BT549 and MDA-MB-231 breast cancer cells in the absence and presence of Cerberus-Fc. Pictures were taken every 5 minutes for approximately 24h using a JuliBr live cell analyzer. (PPTX) [file pone.0114954.s005.pptx]

## Slide 1
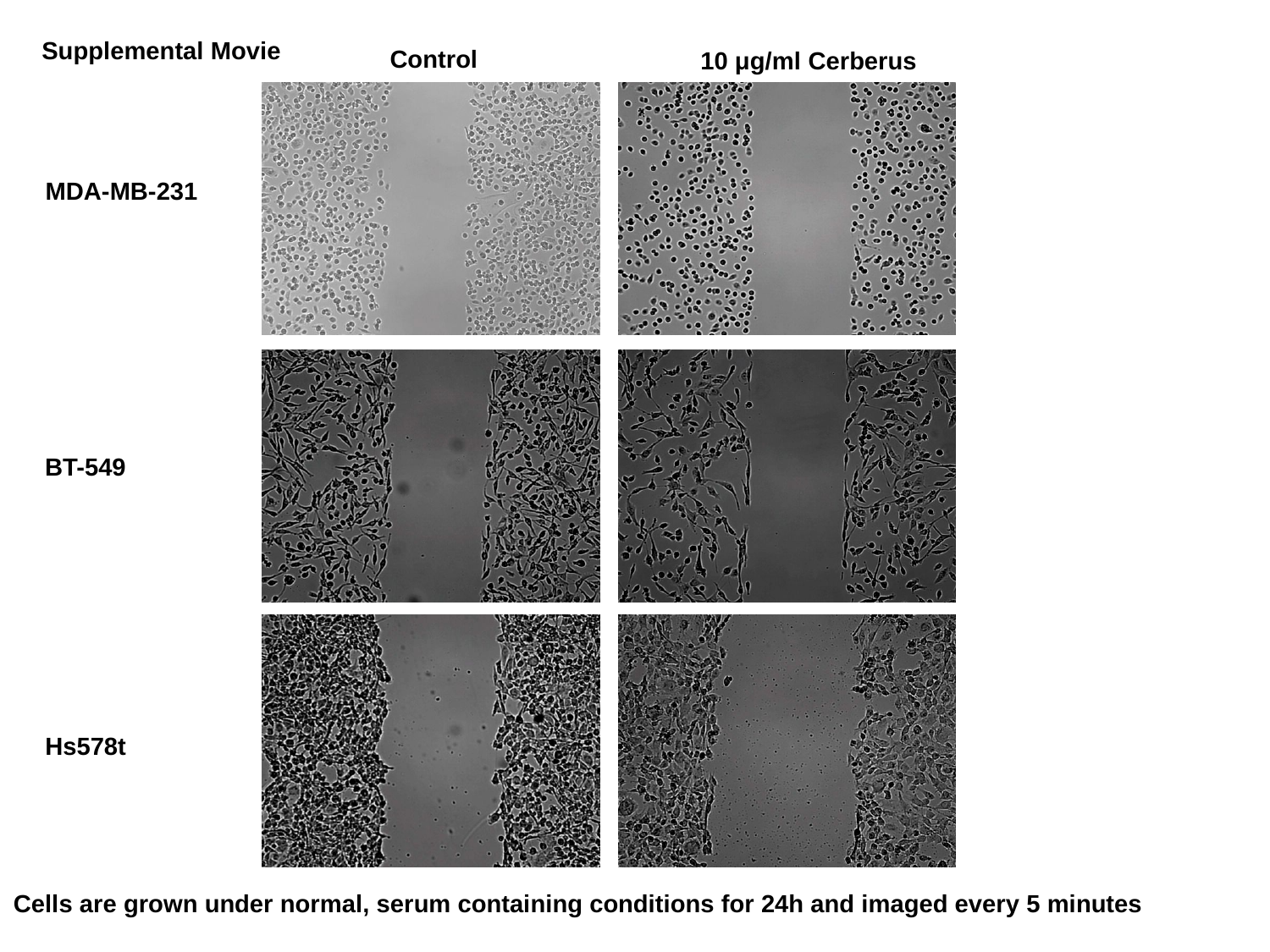

Supplemental Movie
Control
10 μg/ml Cerberus
MDA-MB-231
BT-549
Hs578t
Cells are grown under normal, serum containing conditions for 24h and imaged every 5 minutes
