# Supplementary material for: Human Cerberus Prevents Nodal-Receptor Binding, Inhibits Nodal Signaling, and Suppresses Nodal-Mediated Phenotypes
Source: PLoS One. 2015 Jan 20;10(1):e0114954. doi: 10.1371/journal.pone.0114954 (PMC4300205; doi:10.1371/journal.pone.0114954)
Supplement: S1 Table — (PDF) [file pone.0114954.s006.pdf]

Table S1: Inhibition analysis, manuscript figure 4

Receptor	Inhibitor Concentration	$K_{d(\text{apparent})}$ (M)	$K_{d(\text{calculated})}$ (M) Competitive Model
Cripto-1	40 nM	$2.58e^{-08}$	$5.57e^{-08}$
	400 nM	N.D.	$5.43e^{-07}$
Alk4	40 nM	$1.83e^{-07}$	$4.30e^{-07}$
	400 nM	N.D	$4.20e^{-06}$
ActRIIB	40 nM	$3.00e^{-07}$	$5.60e^{-07}$
	400 nM	N.D.	$5.47e^{-06}$
BMPRII	40 nM	$3.85e^{-09}$	$6.69e^{-09}$
	400 nM	$2.1e^{-08}$	$6.52e^{-08}$
	4000 nM	$1.9e^{-08}$	$6.50e^{-07}$

Apparent K_d measured in the presence of the Cerberus ($K_{d(apparent)}$) approximately obeys the equation for competitive binding, $K_{dapp} = K_d(1 + [\text{Cerberus}]/K_i)$ when comparing binding of Nodal to Cripto, ALK4 and ActRIIB with and without Cerberus. This is not the case for BMPRII. K_d is the dissociation binding constant of the Nodal-receptor interaction in the absence of Cerberus. K_i is the dissociation binding constant of the Nodal-Cerberus interaction.
